# Accuracy of Static Guided Endodontic Access in Pulp Canal Obliteration: Influence of Sleeve Design on Clinical Precision

**DOI:** 10.1016/j.identj.2026.109685

**Published:** 2026-07-31

**Authors:** Anna Muryani, Dudi Aripin, Hendra Dian Adhita Dharsono, Satrio Wicaksono, Zainul Ahmad Rajion, Yurika Ambar Lita, Wandi Prasetia

**Affiliations:** a Conservative Dentistry Department Faculty of Dentistry, Universitas Padjadjaran, Bandung, West Java, Indonesia; b Faculty of Mechanical and Aerospace Engineering, Institut Teknologi Bandung, Bandung, West Java, Indonesia; c Department Oral Maxillofacial Surgery and Oral Diagnosis Kulliyyah of Dentistry International Islamic University Malaysia, Kuantan, Malaysia; d Department Oral Maxillofacial Radiology Faculty of Dentistry, Universitas Padjadjaran, Bandung, West Java, Indonesia

**Keywords:** Pulp canal obliteration, Guided endodontics, CBCT, Access cavity, Clinical accuracy, Endodontic treatment

## Abstract

**Objectives:**

Pulp canal obliteration (PCO) presents a major challenge in endodontic treatment due to canal calcification, which complicates canal localization and increases the risk of errors during freehand access (FHA). Static guided endodontic access (SGEA) has been introduced to improve accuracy; however, the influence of guide sleeve design, particularly material and height, remains unclear.

**Methods:**

This in vitro study used 48 standardized 3D-printed maxillary central incisors with simulated PCO. Tooth-supported guides with titanium, cobalt–chromium (CoCr), and zirconia sleeves at heights of 3, 5, and 7 mm were evaluated, with FHA as control. Pre- and post-operative CBCT scans were superimposed to measure coronal and apical deviation and access cavity volume. Data were analysed using 1-way ANOVA with Bonferroni post hoc tests (α = 0.05).

**Results:**

Guided access showed significantly lower coronal and apical deviations than FHA, especially in labial–lingual directions (*p* < .001). Increased sleeve height improved directional control, with 7 mm sleeves showing the lowest deviations. Sleeve material showed no consistent effect. Guided techniques also enabled more controlled dentin removal.

**Conclusions:**

SGEA improves accuracy and procedural control compared with FHA in simulated PCO. Sleeve height plays a more critical role than material.

**Clinical Relevance:**

Guided endodontics enhances precision, supports conservative dentin removal, and reduces iatrogenic risk, with sleeve height as a key factor.

## Introduction

Pulp canal obliteration (PCO), also referred to as calcific metamorphosis, is a common consequence of dental trauma, particularly in anterior teeth.^1^ The progressive deposition of hard tissue within the root canal system results in narrowing or complete obliteration of the canal space, making endodontic access and canal localization challenging.^1^ When pulpal or periapical pathology develops in such cases, conventional freehand access (FHA) may require excessive dentin removal and is associated with an increased risk of procedural errors, including perforation and canal deviation.^2^, ^3^, ^4^

Static guided endodontic access (SGEA) has been introduced as an alternative approach to improve accuracy in challenging cases such as PCO. This technique combines CBCT imaging and digital planning to guide the bur along a predefined trajectory, facilitating canal localization while potentially reducing iatrogenic complications.^5^^,^^6^ Previous studies have demonstrated that guided endodontics can achieve high accuracy and improve predictability compared with FHA techniques.^2^^,^^3^^,^^6^, ^7^, ^8^

Several technical factors related to guide design may influence the accuracy of guided endodontic procedures, despite these advantages. In guided implant surgery, parameters such as sleeve height, material, and drill sleeve tolerance have been shown to affect drilling stability and accuracy.^4^^,^^9^^,^^10^ Longer sleeves may improve directional control by reducing lateral movement of the bur, whereas variations in material properties, such as hardness and surface characteristics, may influence wear behaviour and guidance precision.^10^

However, the specific influence of sleeve material and height on the three-dimensional accuracy of static guided endodontic access remains insufficiently investigated. Most previous studies have focused on general accuracy outcomes without isolating these design variables. Therefore, a clearer understanding of how sleeve characteristics affect access precision is needed. From a clinical perspective, difficulties in canal localization in pulp canal obliteration often lead to excessive dentin removal, increased risk of perforation, and compromised treatment outcomes. Therefore, improving the precision and predictability of access cavity preparation is critical for successful endodontic management.

The aim of this study was to evaluate the effect of sleeve material and height on the three-dimensional accuracy of static guided endodontic access in a simulated PCO model. The null hypothesis was that sleeve material and sleeve height would have no significant effect on the accuracy of guided endodontic access.

## Materials and methods

**Fig. 1:**
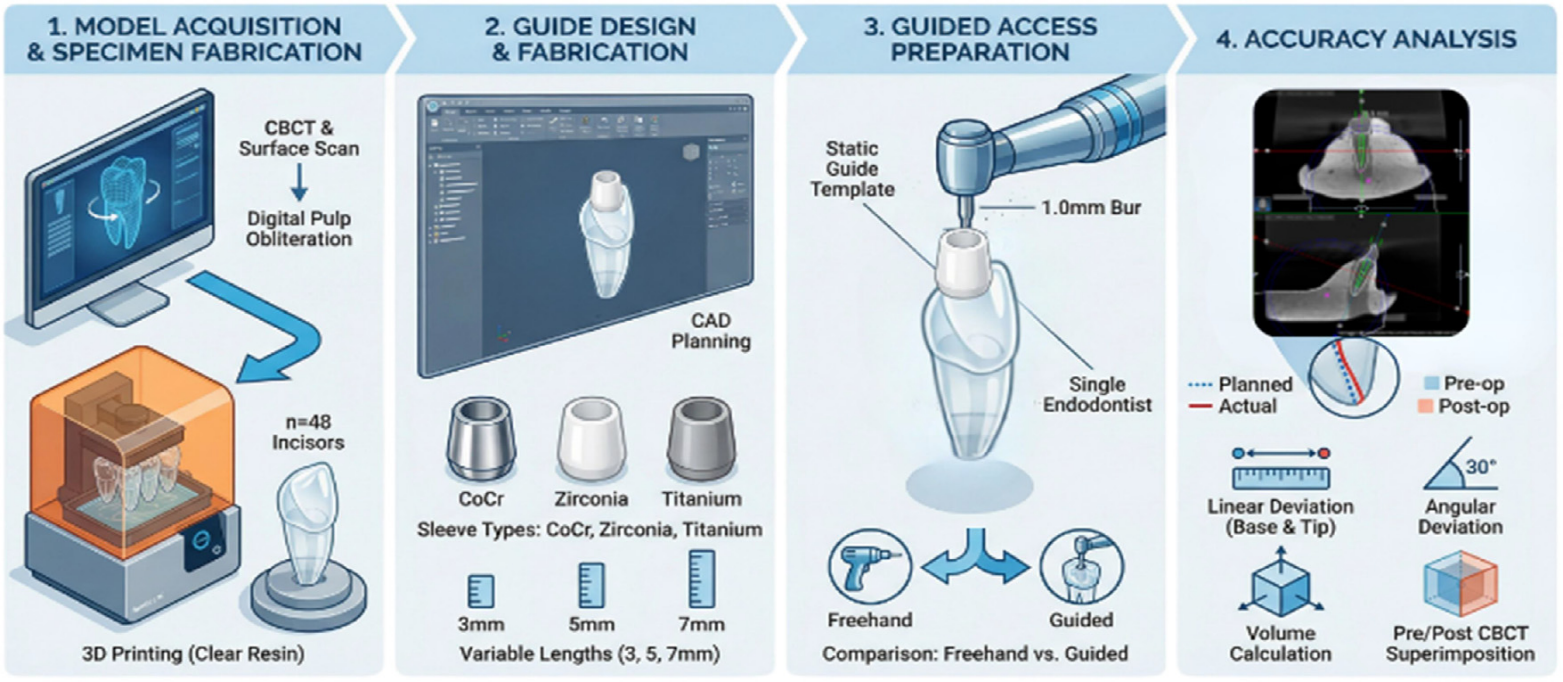
Illustrates the study methodology, including the design, fabrication, and analysis of the custom static guide.

The overall study workflow, including the design, fabrication, guided access procedure, and accuracy analysis of the custom static guide, is illustrated in Figure 1.

### Study design and sample preparation

The experimental setup was designed to replicate clinically relevant conditions of pulp canal obliteration to enhance the translational relevance of the findings. This in vitro study was conducted using 48 standardized 3D-printed replicas of human maxillary central incisors derived from anonymized CBCT datasets. Digital models were modified to simulate pulp canal obliteration and fabricated to ensure consistency across samples. Ethical approval was obtained from the Institutional Review Board of Universitas Padjadjaran (Approval No. 194/UN6.KEP/EC/2025), and no direct human intervention was performed.

A representative tooth was digitized using CBCT imaging (OP300 Maxio, KaVo, Finland; voxel size 0.25 mm) to capture internal morphology, while external surface data were acquired using an intraoral scanner (CEREC Primescan, Dentsply Sirona, Germany). These datasets were integrated to generate the final 3D models. The volumetric DICOM data and surface STL data were co-registered and processed in mesh editing software (Meshmixer, Autodesk, San Rafael). The pulp space was segmented, and a virtual obliteration was modelled in the middle third of the root canal to simulate calcific metamorphosis (pulp canal obliteration). The final phantom geometry was exported as an STL file and fabricated using a stereolithography (SLA) 3D printer (Formlabs 4, Formlabs, Somerville) with clear resin (Young’s modulus ∼2.7–3.0 GPa). This material provided radiopacity and mechanical properties suitable for visualizing the canal and standardizing drilling resistance.

Specimens were embedded in a dental arch simulator (Trimunt ARISTA TA-500) with a 2-mm resin base to replicate clinical alveolar orientation. A baseline pre-operative CBCT scan was acquired for each mounted specimen (OP300 Maxio; 8 × 15 cm FOV, 0.25 mm voxel size, 90 kVp, 3.2–12.5 mA) to serve as the reference dataset for planning.

### Guide design and fabrication

**Fig. 2:**
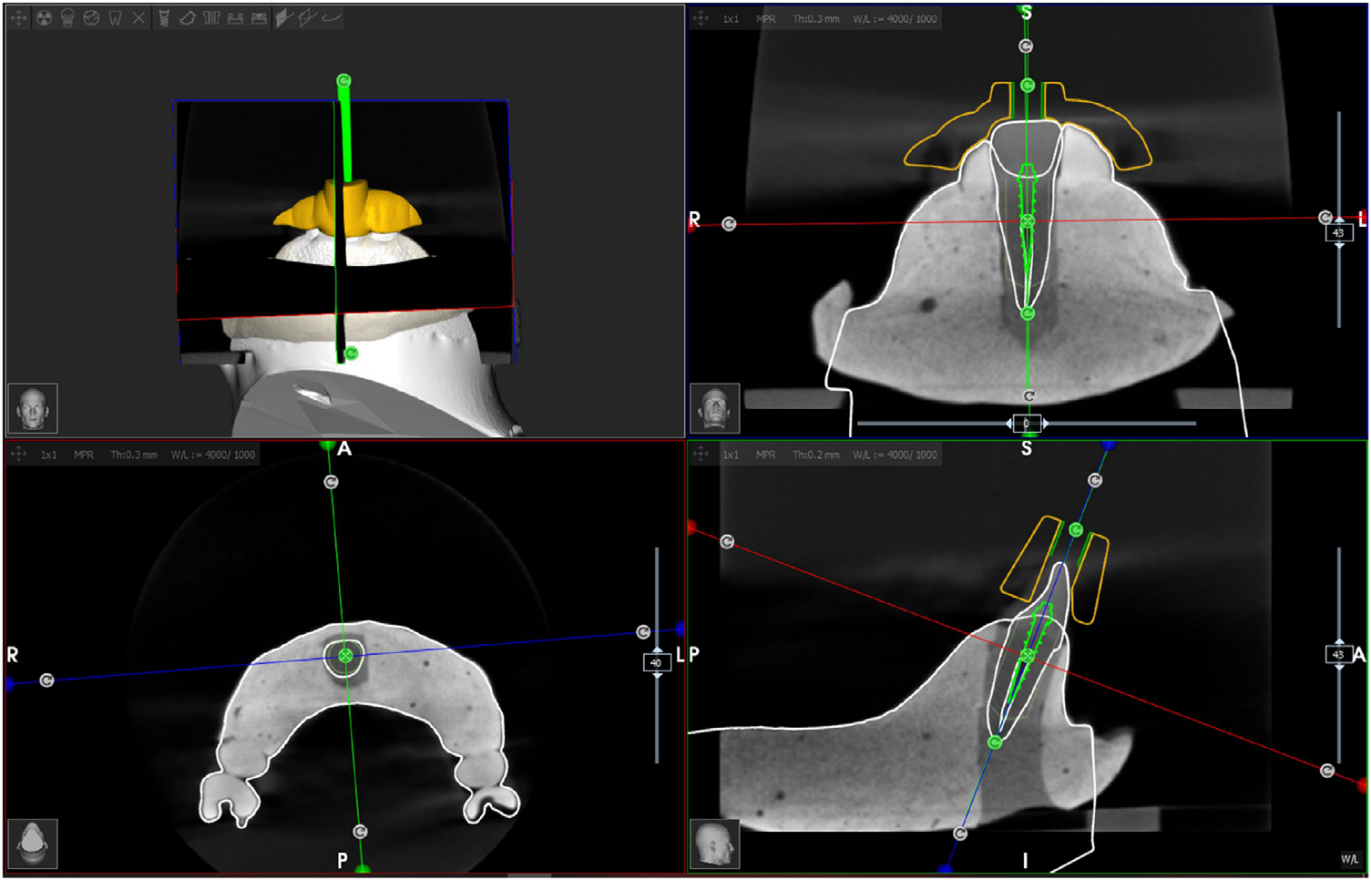
Custom static guides design planning using AIS 3D App, Acteon Group, Italy.

Virtual planning was performed using implant planning software (Acteon Imaging Suite [AIS] 3D App, Acteon Group). The access trajectory was planned to align coaxially with the canal orifice and extend to the apical patent segment. Custom static guides were designed to fit the arch model and incorporate specific sleeve geometries (Figure 2).

To evaluate the impact of guidance variables, guides were designed to accommodate sleeves of varying materials and heights. Guide sleeves were fabricated using three different materials. Cobalt–chromium (CoCr) sleeves were manufactured by selective laser melting (SLM) using a metal 3D printing system (HBD 150/150D) with CoCr powder (Starbond CoS Powder 30). Zirconia (ZrO₂) sleeves were produced through a CAD/CAM milling process using zirconia blanks (IPS e.max ZirCAD, Ivoclar) and a milling unit (CEREC, Dentsply Sirona). Titanium (Ti) sleeves served as the control group and consisted of prefabricated guide sleeves with a standardized height of 5 mm (StecoGuide, Steco-System Technik).

### Experimental groups and guide design

All sleeves were designed with an internal diameter of 1.0 mm to match the endodontic bur. To evaluate the effect of sleeve height, CoCr and zirconia sleeves were fabricated at 3 heights (3, 5, and 7 mm). Specimens were randomly allocated into eight groups (n = 6): FHA control, titanium control (5 mm), CoCr (3, 5, 7 mm), and zirconia (3, 5, 7 mm). Guides were 3D-printed in clear resin, and sleeves were secured to ensure coaxial alignment with the planned drilling trajectory. The FHA group was subdivided according to standardized drilling depths corresponding to the sleeve heights (3, 5, and 7 mm) to enable consistent comparison across groups.

### Guided access protocol

A single calibrated endodontist performed all access preparations to minimize inter-operator variability. For guided groups, a 1.0 mm endodontic bur (Steco-System Technik) was operated at 700–1000 rpm (approx. 2.5 N·cm torque) under continuous saline irrigation and advanced to the planned working length constrained by the sleeve. The FHA control group was treated using the same bur and angulation based on the planned entry point, utilizing a dental operating microscope for visualization.

### Radiographic image analysis

Following preparation, a post-operative CBCT scan was acquired with parameters identical to those of the pre-operative baseline scan to ensure image compatibility.

### Image registration and superimposition

Quantitative analysis was performed within the Acteon Imaging Suite (AIS). The pre-operative (planning) and post-operative (result) DICOM datasets were spatially registered. A rigid, point-based registration algorithm was utilized, employing three stable anatomical landmarks: the incisal edge, the cervical margin, and the root apex. Superimposition accuracy was visually verified in orthogonal planes prior to measurement.

### Deviation measurements

**Fig. 3:**
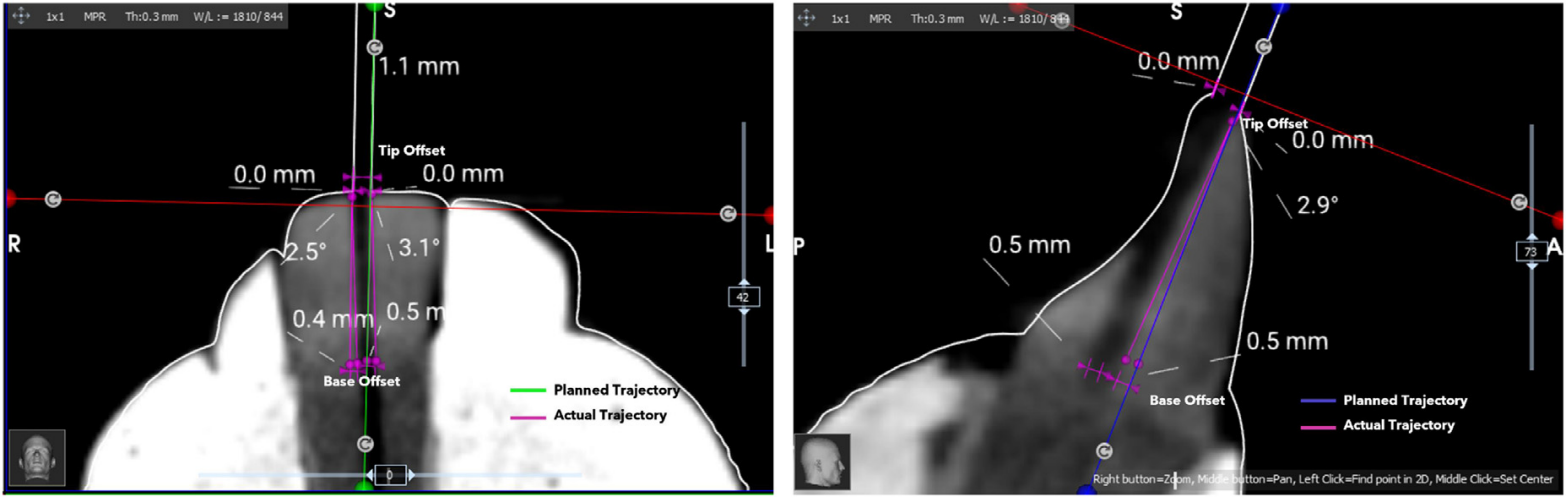
Deviation measurement between the planned virtual trajectory and the actual prepared canal path using AIS 3D App, Acteon Group, Mt. Laurel, NJ, USA.

Deviations between the planned virtual trajectory and the actual prepared canal path were calculated using geometric analysis. Linear deviation was defined as the perpendicular Euclidean distance between the central axes of the planned and prepared paths. Measurements were performed at two reference levels: the base offset at the coronal entry point and the tip offset at the endpoint of the preparation. For directional analysis, deviations were further resolved into mesial, distal, labial, and lingual components (Figure 3).

### Volumetric analysis

**Fig. 4:**
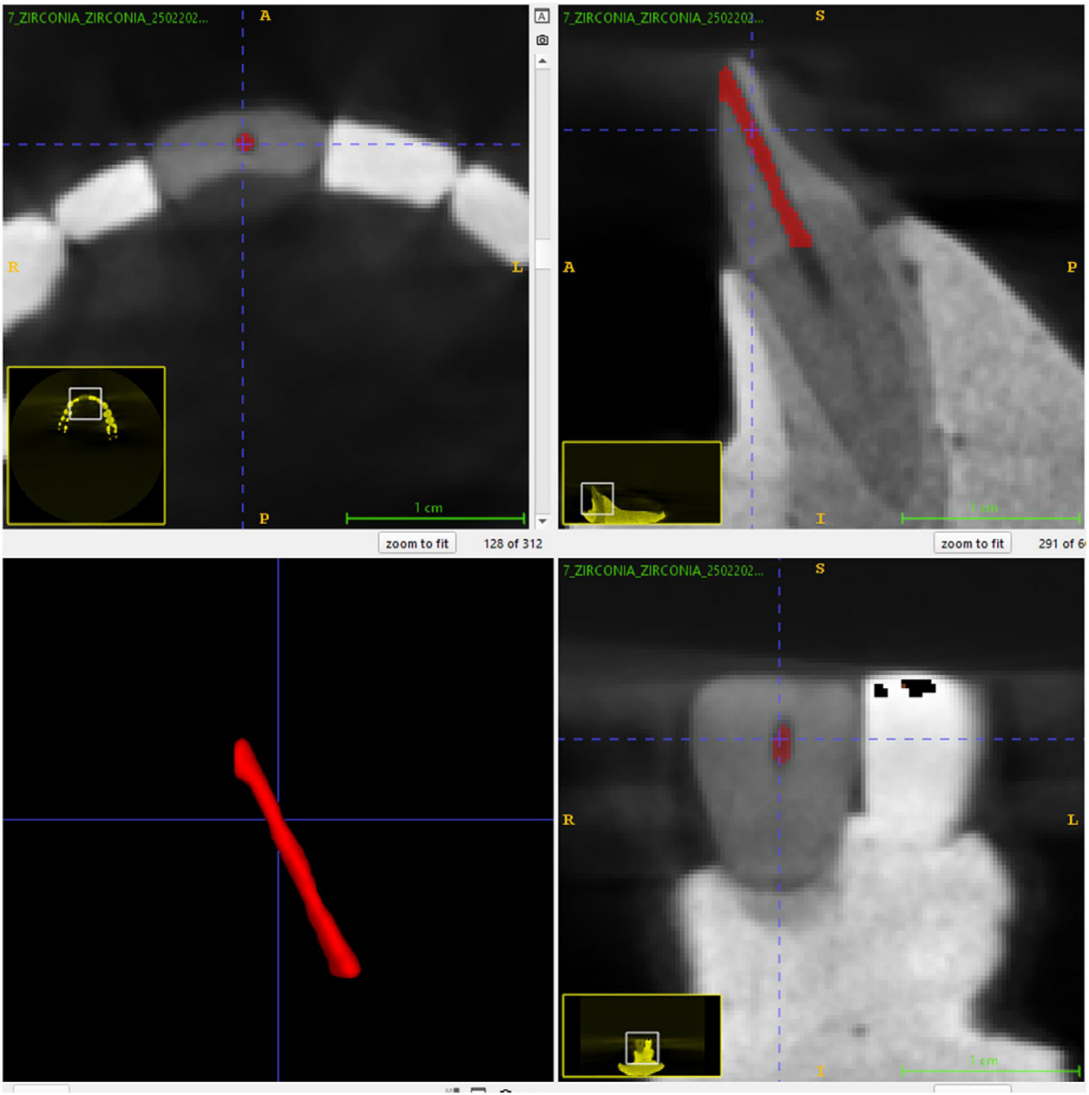
Volumetric measurement using ITK SNAP version 4.2.2.

Volumetric analysis was performed to quantify hard tissue removal using semi-automatic segmentation of pre- and post-operative CBCT datasets. The volume of removed hard tissue was calculated as the difference between the preoperative canal space and the post-operative prepared cavity (Figure 4).

All measurements were conducted using CBCT image superimposition and three-dimensional analysis software. Each measurement was performed twice by the same operator with a two-week interval, and the mean values were used for analysis.

### Statistical analysis

All measurements were performed by two independent, blinded examiners calibrated to the software. Inter- and intra-examiner reliability were assessed using the Intraclass Correlation Coefficient (ICC), with values >0.90 considered excellent.

Data were analysed using statistical software (SPSS). Normality was assessed using the Shapiro–Wilk test. Differences in linear deviation and volumetric changes were analysed using one-way analysis of variance (ANOVA), followed by Bonferroni post hoc tests. The level of significance was set at α = 0.05.

## Results

### Base offset

**Fig. 5:**
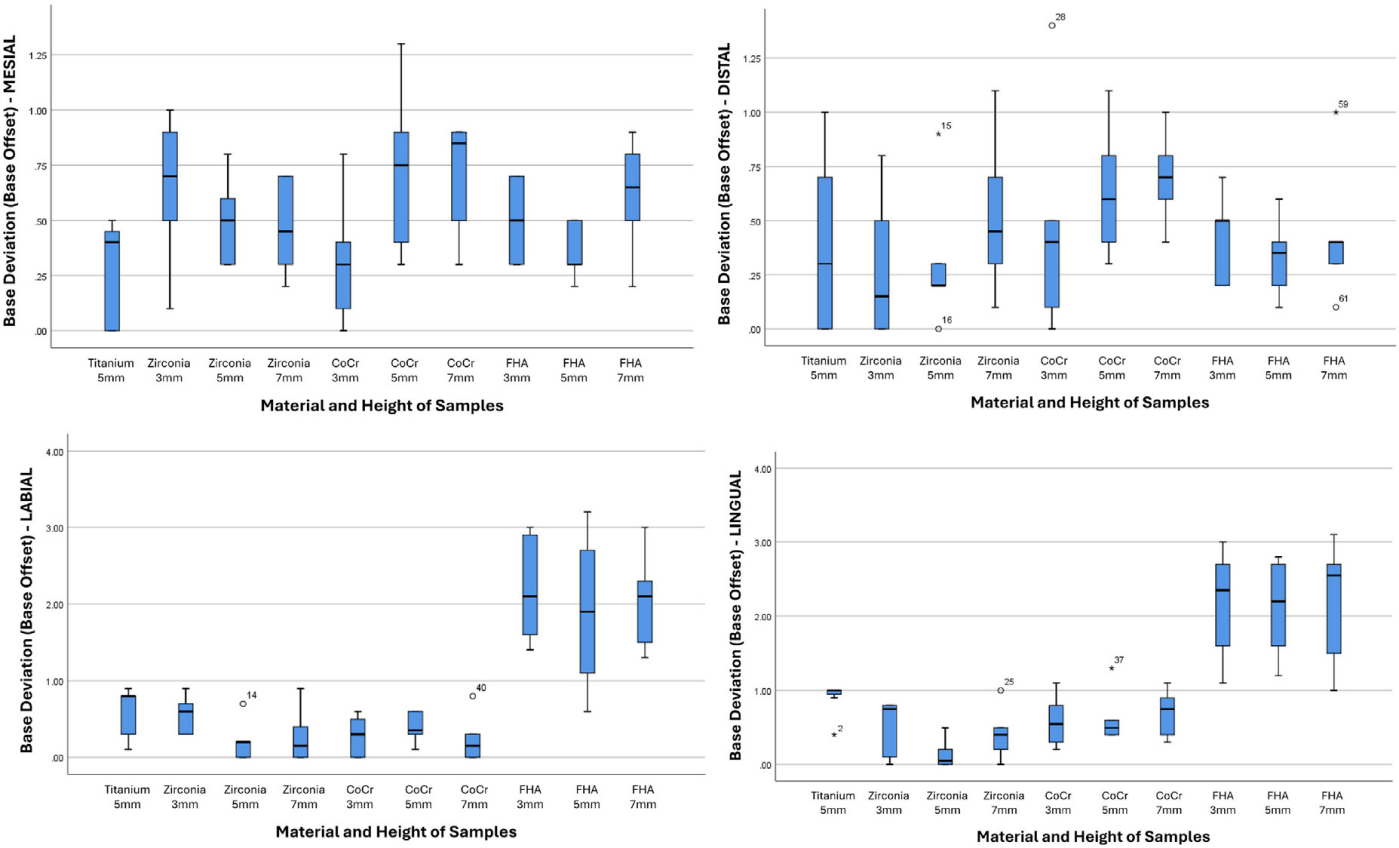
Mean base offset values (mm) in the mesial, distal, labial, and lingual directions for all experimental groups. Data are presented as mean ± SD, comparing different sleeve heights (3 mm, 5 mm, and 7 mm) and sleeve materials (Titanium, CoCr, and Zirconia).

The mean base offset values for all groups are presented in Table 1 and Figure 5. Significant differences were observed among groups in the mesial (*p* = .012), labial (*p* < .001), and lingual directions (*p* < .001), while no significant difference was found in the distal direction (*p* = .394).

**Table 1:** Descriptive statistics of base offset (mm) in the mesial, distal, labial, and lingual directions for all experimental groups.

			Titanium 5 mm (n = 6)	Zirconia 3 mm (n = 6)	Zirconia 5 mm (n = 6)	Zirconia 7 mm (n = 6)	CoCr 3 mm (n = 6)	CoCr 5 mm (n = 6)	CoCr 7 mm (n = 6)	FHA 3 mm (n = 6)	FHA 5 mm (n = 6)	FHA 7 mm (n = 6)
**Base deviation (base offset)**	**Mesial**	**Mean ± SD**	0.25 ± 0.24	0.65 ± 0.32	0.50 ± 0.20	0.46 ± 0.20	0.31 ± 0.28	0.73 ± 0.36	0.71 ± 0.25	0.50 ± 0.21	0.35 ± 0.12	0.61 ± 0.24
		**Median (Min-Max)**	0.40 (0.00-0.50)	0.70 (0.10-1.00)	0.50 (0.30-0.80)	0.45 (0.20-0.70)	0.30 (0.00-0.80)	0.75 (0.30-1.30)	0.85 (0.30-0.90)	0.50 (0.30-0.70)	0.30 (0.20-0.50)	0.65 (0.20-0.90)
		*p****-value***	**<.001**									
											
	**Distal**	**Mean ± SD**	0.38 ± 0.44	0.26 ± 0.33	0.30 ± 0.30	0.51 ± 0.34	0.46 ± 0.49	0.63 ± 0.29	0.70 ± 0.20	0.43 ± 0.19	0.33 ± 0.17	0.43 ± 0.30
		**Median (Min-Max)**	0.30 (0.00-1.00)	0.15 (0.00-0.80)	0.20 (0.00-0.90)	0.45 (0.10-1.10)	0.40 (0.00-1.40)	0.60 (0.30-1.10)	0.70 (0.40-1.00)	0.50 (0.20-0.70)	0.35 (0.10-0.60)	0.40 (0.10-1.00)
		*p****-value***	**<.001**									
											
	**Labial**	**Mean ± SD**	0.57 ± 0.34	0.56 ± 0.23	0.21 ± 0.25	0.26 ± 0.34	0.28 ± 0.25	0.38 ± 0.19	0.23 ± 0.31	2.18 ± 0.70	1.90 ± 0.98	2.05 ± 0.61
		**Median (Min-Max)**	0.80 (0.10-0.90)	0.60 (0.30-0.90)	0.20 (0.00-0.70)	0.15 (0.00-0.90)	0.30 (0.00-0.60)	0.35 (0.10-0.60)	0.15 (0.00-0.80)	2.10 (1.40-3.00)	1.90 (0.60-3.20)	2.10 (1.30-3.00)
		*p***-value**	**<.001**									
											
	**Lingual**	**Mean ± SD**	0.90 ± 0.22	0.53 ± 0.37	0.13 ± 0.19	0.41 ± 0.33	0.58 ± 0.33	0.61 ± 0.34	0.70 ± 0.31	2.18 ± 0.74	2.11 ± 0.64	2.23 ± 0.80
		**Median (Min-Max)**	1.00 (0.40-1.00)	0.75 (0.00-0.80)	0.05 (0.00-0.50)	0.40 (0.00-1.00)	0.55 (0.20-1.10)	0.50 (0.40-1.30)	0.75 (0.30-1.10)	2.35 (1.10-3.00)	2.20 (1.20-2.80)	2.55 (1.00-3.10)
		*p***-value**	**<.001**									

* The analysis of mean differences for normally distributed numerical data was performed using a one-way ANOVA with a significance level of *p* < .05. A Bonferroni post hoc test was conducted when *p* < .05.

FHA groups demonstrated higher deviations, particularly in the labial and lingual directions (approximately 1.90-2.23 mm), compared with guided groups, which showed lower values (approximately 0.13-0.57 mm). Among guided groups, zirconia sleeves with 5 mm and 7 mm heights exhibited lower deviations in the labial-lingual direction.

### Tip offset

**Fig. 6:**
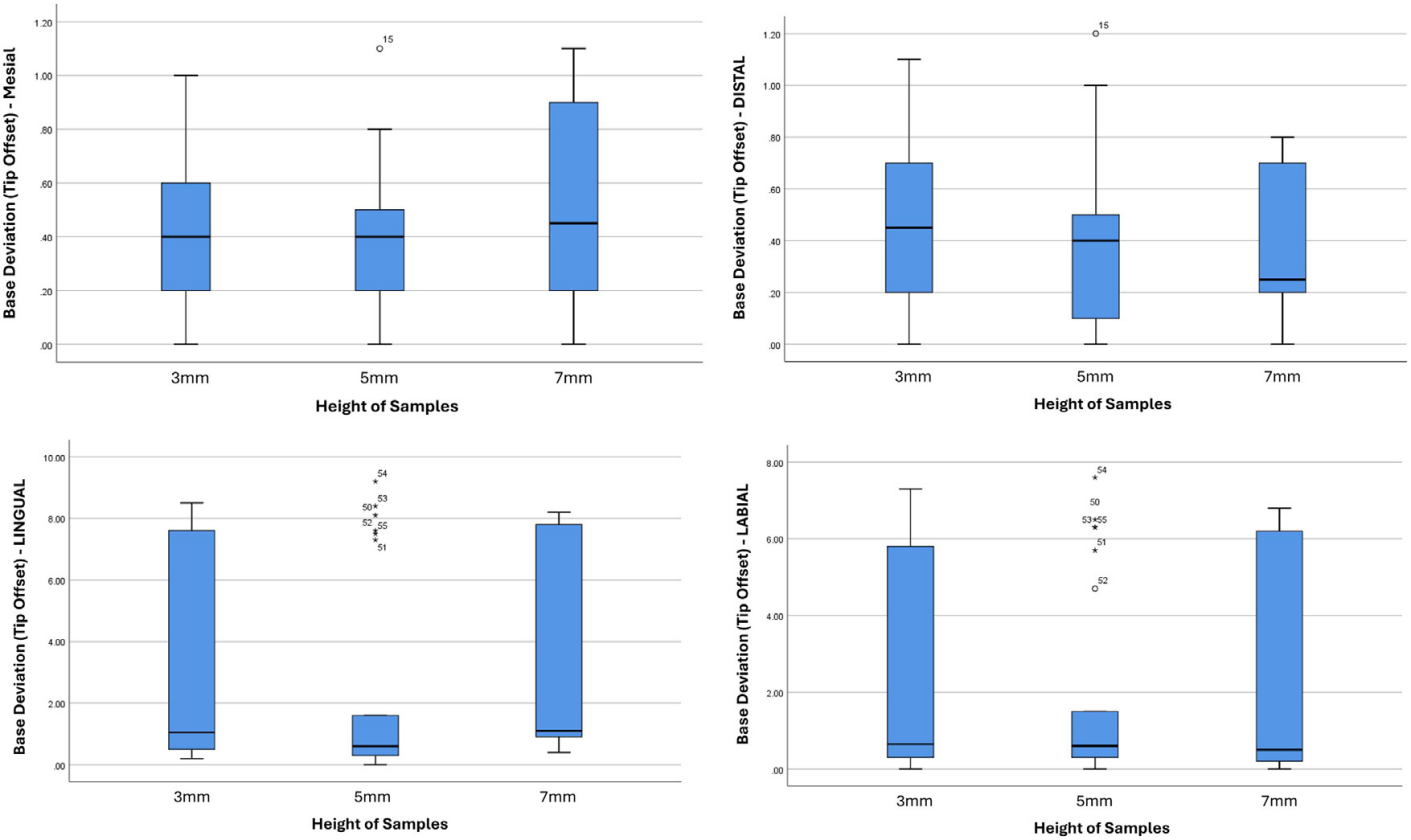
Comparison of tip offset (mm) among experimental groups according to sleeve height (3 mm, 5 mm, and 7 mm) and sleeve material (Titanium, CoCr, and Zirconia). Values are expressed as mean ± SD.

Tip offset values are summarized in Table 2 and Figure 6. Significant differences were observed in the mesial, labial, and lingual directions (*p* < .001), whereas no significant difference was found in the distal direction (*p* = .138).

**Table 2:** Descriptive statistics and one-way ANOVA results for tip offset (mm) according to sleeve height and sleeve material.

			Titanium 5 mm (n = 6)	Zirconia 3 mm (n = 6)	Zirconia 5 mm (n = 6)	Zirconia 7 mm (n = 6)	CoCr 3 mm (n = 6)	CoCr 5 mm (n = 6)	CoCr 7 mm (n = 6)	FHA 3 mm (n = 6)	FHA 5 mm (n = 6)	FHA 7 mm (n = 6)
Base deviation (tip offset)	Mesial	Mean ± SD	0.1857 ± 0.19518	0.6833 ± 0.23166	0.4667 ± 0.42740	0.2500 ± 0.21679	0.2333 ± 0.26583	0.5167 ± 0.14720	0.9500 ± 0.23452	0.3833 ± 0.26394	0.4833 ± 0.14720	0.3833 ± 0.28577
		Median (Min-Max)	0.10 (0.00-0.50)	0.65 (0.40-1.00)	0.50 (0.00-1.10)	0.30 (0.00-0.50)	0.20 (0.00-0.60)	0.50 (0.40-0.80)	1.05 (0.50-1.10)	0.30 (0.10-0.80)	0.45 (0.30-0.70)	0.35 (0.00-0.80)
		*p-value*	<.001									
											
	**Distal**	**Mean ± SD**	0.2571 ± 0.26992	0.7333 ± 0.24221	0.4667 ± 0.45461	0.2000 ± 0.27568	0.4000 ± 0.39497	0.4500 ± 0.05477	0.5000 ± 0.33466	0.2833 ± 0.11690	0.4667 ± 0.37238	0.3500 ± 0.20736
		**Median (Min-Max)**	0.20 (0.00-0.60)	0.70 (0.40-1.10)	0.45 (0.00-1.20)	0.10 (0.00-0.70)	0.35 (0.00-0.90)	0.45 (0.40-0.50)	0.60 (0.00-0.80)	0.25 (0.20-0.50)	0.40 (0.10-1.00)	0.25 (0.20-0.70)
		*p****-value***	**<.001**									
											
	**Labial**	**Mean ± SD**	1.05 ± 0.33	0.30 ± 0.08	0.10 ± 0.24	0.56 ± 0.42	0.55 ± 0.35	0.45 ± 0.16	0.18 ± 0.22	6.3 ± 0.69	6.18 ± 0.95	6.23 ± 0.60
		**Median (Min-Max)**	1.00 (0.60-1.50)	0.30 (0.20-0.40)	0.00 (0.00-0.60)	0.45 (0.10-1.30)	0.65 (0.00-0.90)	0.50 (0.20-0.60)	0.10 (0.00-0.50)	6.45 (5.30-7.30)	6.30 (4.70-7.60)	6.30 (5.10-6.80)
		*p***-value**	**<.001**									
											
	**Lingual**	**Mean ± SD**	1.02 ± 0.55	0.75 ± 0.33	0.13 ± 0.16	1.06 ± 0.42	0.98 ± 1.10	0.46 ± 0.12	0.85 ± 0.24	7.78 ± 0.68	8.01 ± 0.70	7.81 ± 0.51
		**Median (Min-Max)**	1.10 (0.30-1.60)	0.85 (0.20-1.10)	0.10 (0.00-0.40)	1.10 (0.40-1.70)	0.55 (0.20-3.10)	0.45 (0.30-0.60)	0.95 (0.50-1.10)	7.80 (6.60-8.50)	7.85 (7.30-9.20)	8.00 (6.80-8.20)
		*p***-value**	**<.001**									

* The analysis of mean differences for normally distributed numerical data was performed using a one-way ANOVA with a significance level of *p* < .05. A Bonferroni post hoc test was conducted when *p* < .05.

FHA groups showed substantially higher deviations in the labial and lingual directions (approximately 6-8 mm cumulative deviation values) compared with guided groups, which demonstrated lower values (approximately 0.10-1.05 mm). The lowest deviations were observed in the zirconia 5 mm group.

### Access cavity volume

**Fig. 7:**
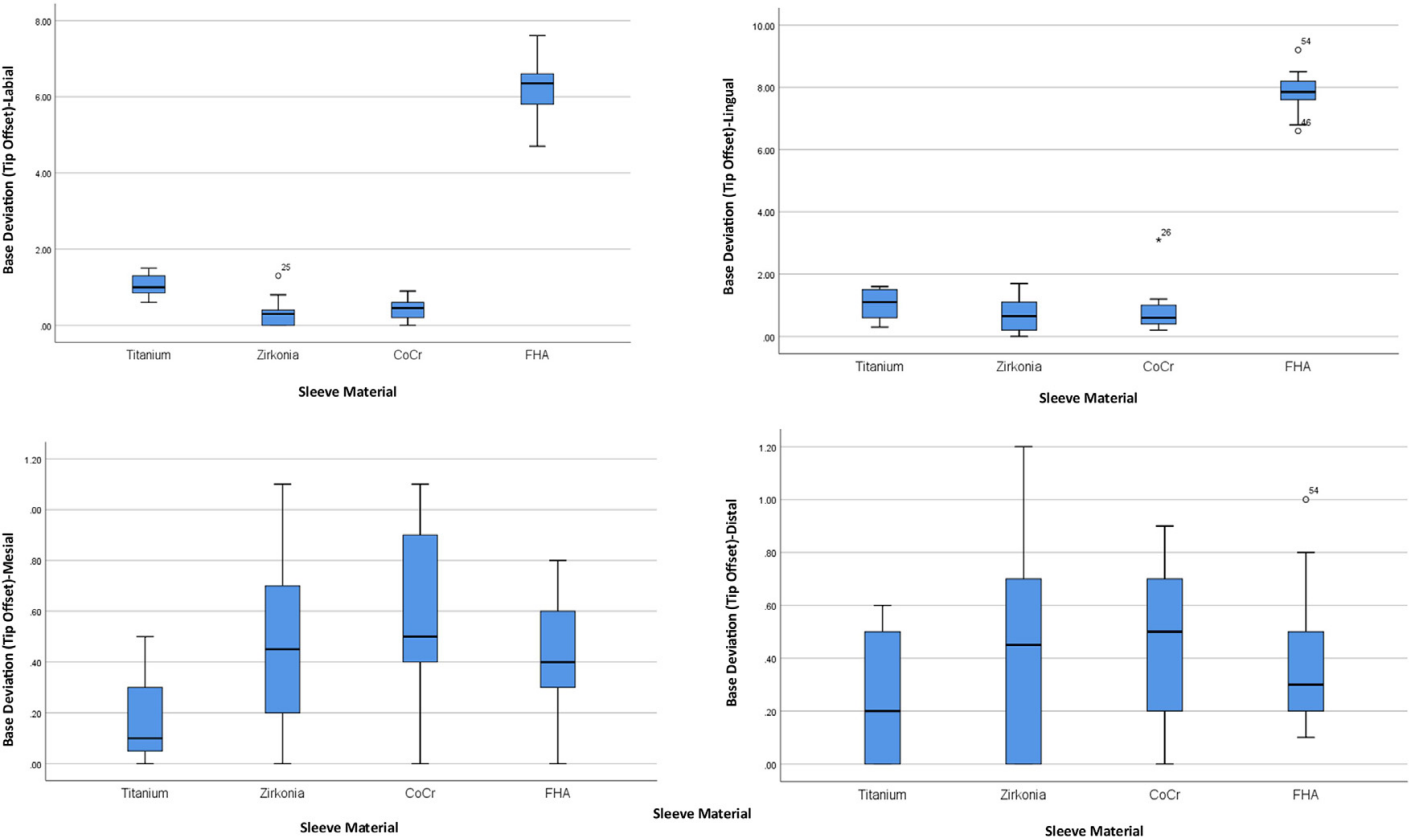
Final access cavity volume (mm³) for each experimental group. Bars represent mean ± SD for different sleeve heights and materials used during guided endodontic access preparation.

Access cavity volumes are presented in Table 3 and Figure 7. A significant difference among groups was observed (*p* = .026). Higher volumes were observed in metallic sleeve groups, particularly CoCr and titanium (approximately 13–16 mm³), whereas lower volumes were found in zirconia and FHA groups (approximately 8–11 mm³). The lowest mean volume was observed in the FHA 3 mm group.

**Table 3:** Descriptive statistics and statistical comparison of access cavity volume (mm³) among experimental groups.

VOLUME	Titanium 5 mm (n = 6)	Zirconia 3 mm (n = 6)	Zirconia 5 mm (n = 6)	Zirconia 7 mm (n = 6)	CoCr 3 mm (n = 6)	CoCr 5 mm (n = 6)	CoCr 7 mm (n = 6)	FHA 3 mm (n = 6)	FHA 5 mm (n = 6)	FHA 7 mm (n = 6)
**Mean ± SD**	15.20 ± 4.10	14.17 ± 3.99	9.33 ± 1.90	11.19 ± 2.87	15.00 ± 4.71	13.41 ± 8.15	16.26 ± 6.33	8.60 ± 1.63	9.86 ± 4.28	9.20 ± 4.74
**Median (Min-Max)**	13.67 (12.52-24.06)	13.24 (9.02-20.77)	9.345 (6.67-11.84)	11.71 (7.22-14.22)	15.21 (9.08-23.09)	10.01 (7.90-29.38)	16.215 (6.55-25.30)	9.21 (6.41-10.27)	10.02 (4.81-16.13)	8.22 (4.81-16.13)
*p****-value***	**<.001**									

* The analysis of mean differences for normally distributed numerical data was performed using a one-way ANOVA with a significance level of *p* < .05. A Bonferroni post hoc test was conducted when *p* < .05.

## Discussion

The present in vitro study demonstrated that static guided endodontic access resulted in lower deviation and reduced access cavity volume compared with the FHA approach. These findings are consistent with previous studies reporting that guided endodontic techniques improve accuracy and reduce procedural errors, particularly in cases involving pulp canal obliteration.^2^^,^^6^^,^^11^

The clinical relevance of these findings lies in the potential to improve safety and precision during access cavity preparation in teeth with pulp canal obliteration. By reducing deviation and preserving dentin, guided endodontic techniques may help minimize iatrogenic complications and support more conservative treatment approaches in daily clinical practice.

The significantly higher deviations observed in the FHA group can be explained by the absence of mechanical guidance during drilling. In FHA procedures, the operator relies primarily on visual and tactile feedback, which increases the likelihood of angular deviation and lateral drift. In contrast, static guides provide a predefined drilling trajectory that enhances canal localization and minimizes unnecessary dentin removal.^11^^,^^12^

The influence of sleeve height observed in this study supports previous findings that increased sleeve length improves drilling stability and accuracy. Longer sleeves may reduce lateral play between the bur and guide, thereby enhancing alignment along the planned trajectory.^10^

Although sleeve material did not consistently affect linear deviation in this study, differences in material properties such as surface roughness, wear resistance, frictional behaviour, and thermal conductivity may still influence guidance precision and drilling performance.^5^^,^^10^ Previous studies have shown that sleeve material can affect frictional contact and heat generation during guided endodontic access, which may indirectly influence drilling stability and accuracy.^13^

For example, zirconia sleeves, due to their low thermal conductivity, tend to concentrate heat locally, whereas metallic sleeves such as cobalt–chromium may allow more efficient heat dissipation.^13^ These differences in thermal and mechanical behaviour may contribute to variations in drill sleeve interaction and overall guidance performance.

However, the large deviations observed in the FHA group, particularly in the labial and lingual directions, should be interpreted with caution. These values may reflect cumulative angular deviation along the drilling path rather than true apical displacement. In addition, variations in measurement methodology may contribute to overestimation of tip deviation values.

The accuracy of CBCT-based measurements is inherently influenced by voxel size, image resolution, and image registration processes.^14^, ^15^, ^16^ In the present study, a voxel size of 0.25 mm was used, which limits the ability to detect very small dimensional differences. Therefore, measurements approaching or below this threshold should be interpreted as approximate rather than exact values.^14^ Furthermore, CBCT has lower spatial resolution compared with micro-CT, which may affect the precision of three-dimensional deviation analysis.^15^

Another important limitation is the use of standardized 3D-printed resin models. Although this approach allows for controlled experimental conditions and high reproducibility, resin materials do not fully replicate the mechanical properties and anatomical variability of natural dentin. As a result, the findings of this study may not be directly transferable to clinical conditions, where additional factors such as patient movement, limited access, and operator variability may influence accuracy.^14^^,^^15^

In addition, the subdivision of the FHA group in this study was based on standardized drilling depth corresponding to the sleeve height in guided groups, allowing consistent comparison across experimental conditions. However, this grouping does not represent a true clinical scenario and should be interpreted within the context of experimental standardization.

Within these limitations, the present findings suggest that guide design parameters, particularly sleeve height, may influence the accuracy of static guided endodontic access. These results support the concept that improved mechanical guidance can enhance procedural precision and contribute to minimally invasive endodontic approaches. However, further studies, especially ex vivo and clinical investigations, are required to validate these findings and determine their clinical applicability. Overall, static guided endodontic access demonstrated improved accuracy and more conservative cavity preparation compared with FHA techniques under controlled in vitro conditions.

## Conclusions

Within the limitations of this in vitro study, static guided endodontic access generally demonstrated lower deviation and reduced cavity volume compared with the FHA approach. Sleeve height appeared to influence accuracy, with longer sleeves showing improved directional control. However, these findings should be interpreted cautiously and require further validation in clinical settings.

## Funding

This research was partially funded by Research Funder, Direktorat Riset dan Pengabdian Masyarakat Universitas Padjadjaran (DRPM UNPAD).

## Author contributions

Conceptualization, A.M.; methodology, A.M., D.A.,and S.W; validation, A.M, H.D.A.D., and Z.A.R.; formal analysis, AM, W.P.; software, A.M., Y.A.L., and W.P.; supervision, D.A., H.D.A.D., S.W.,. and Z.A.R.; writing original draft, A.M.; writing review and editing, Y.A.L., and W.P. All authors have read and agreed to the published version of the manuscript.

## Declaration of competing interest

The authors declare the following financial interests/personal relationships which may be considered as potential competing interests: Anna Muryani reports financial support was provided by Padjadjaran University. Anna Muryani reports a relationship with Padjadjaran University that includes: employment and funding grants. If there are other authors, they declare that they have no known competing financial interests or personal relationships that could have appeared to influence the work reported in this paper.
